# Case of Hydrophobia

**Published:** 1845-07

**Authors:** 


*Case of Hydrophobia.*—The following account of a case of this terrible disease, which recently occurred in this city, is given from notes which have been obligingly furnished by Dr. James P. White, the attending Physician.

The subject was Robert Ferguson, from Ireland, a blacksmith: aged twenty-eight; had been a resident of Buffalo fifteen months: and during this time had had two or three attacks of intermittent fever of an ordinary character, for which he was attended by Dr. White.

April 21st. He sent word to Dr. W. that he had a recurrence of his old affection, (intermittent fever,) having experienced a distinct chill on that day; and that he had pain and numbness in his left arm. He wished Dr. W. to send him some medicine. Dr. W. prescribed calomel and rhubarb aa grs. x. On the 22d, A. M., Dr. W. was requested to visit him. The pulse was found to be 100, and rather hard. No circumstances then indicated the specific nature of the disease. It was supposed to be, as the patient supposed, an attack of intermittent fever, with an unusual protraction of the hot stage, and accompanied with neuralgia of the left arm. The cathartic taken the previous night had not operated, and Ol. Ricini was directed.

The patient was seen in the afternoon by Dr. Clarke, who bled him ℥xxx, and applied sinapisms to shoulder and spine. At this visit Dr. Clarke ascertained, that upon his attempting to take the oil in the forenoon, he was seized with a peculiar and distressing paroxysm. The oil was prescribed to him in water, and he was sitting on the side of the bed; on taking it into his mouth, a small portion only, if any, was swallowed, the remainder was ejected with force; and, at the same time, the patient, starting up from the bed, his countenance expressing remarkable agitation, and all the agony of suffocation, rushed with violence around the room, and, at length, plunged into the bed of a fellow invalid in another part of the room. He said the oil produced a distress, which impelled him to act as he did, and that he could not restrain himself.

23d, A. M., Dr. White being absent from town, he was again seen by Dr. Clarke,—Found he had been quiet for the most part during the night, but had not slept. Had made efforts to drink, but was unable. The attempt excited violent paroxysms, analogous to that of the preceding day, excited by the oil. Dr. C. diagnosticated Hydrophobia, and associated with him Dr. Wilcox. He had taken during the night, every four hours, powders composed of P. Dovers, grs. vi.; Proto chlor. Hyd. grs. i.; Tart ant. et Potas. grs. 1-6. These were taken mixed with a little stewed currants; and were swallowed with considerable difficulty, by taking into the mouth very small portions at a time. There seemed to be a difficulty in bringing the morsels into the mouth. He would make several efforts before he succeeded, and, sometimes, give over the attempt, declaring that it was impossible. The difficulty appeared to consist in a premonition of laryngeal spasms whenever the attempt was made. When he, at length, introduced a portion into the mouth, he did so with a nervous rapidity, and, at the same time, made a quick and vehement effort of deglutition.

These powders were continued until noon, when he was cupped freely over the cervical vertebræ; and the following prescriptions ordered; ℞. Ext. Stram. ʒi.; S. Morph. grs. ii.; M. f. pil. 12. One to be taken from one to four hours. ℞. Proto. chlor. Hyd. grs. ii.; Tart. ant. grs. 1-6; M. f. pil. 1. To be taken alternately with the preceding. At 5, P. M., seen by Dr. White, who confirmed the diagnosis. In the evening Dr. Hamilton was called in consultation. It was now ascertained that on the 15th of March last, he was bitten on the metacarpo-phalangeal articulation of the little finger, by a small dog, which came pursuing a cat into a house where he was sitting. He attempted to lay his hand on the dog's tail, when the dog turned, and bit him in two or three places, as just mentioned, and immediately disappeared, nor could he discover afterward whither he went, or whence he came. The wounds were about ten days in healing. It was, also, ascertained that for a week previous to the present attack, he had had pain between the shoulders, and in the arm. On the day before he applied for medicine, (21st,) which was Sunday, his arm and hand felt very cold, and painful, so that he remained at home, keeping the extremity warmly covered, the whole day. There was pain, also, in the axilla, and its neighbourhood. On the 21st, he went to work as usual, and continued working until noon, when the chill occurred, with increase of pain, and he went to bed.

It is important to remark, that although the patient answered various inquiries relative to the circumstances attending the wound, he did not suspect that any importance was attached to it, excepting as accounting for the pain in the arm. He persisted in referring the origin of his spasms to the oil, in connection with which they first appeared. He declared that it poisoned him, and, at his desire, it was examined, and found to have been taken from a bottle, the greater portion of the contents of which had been administered with no ill effects. The name of Hydrophobia was not mentioned to him, and he evidently had no idea of the nature of the disease under which he was labouring.

Dr. White states that the paroxysms were excited, at first, by the attempt to drink; afterward, by the sight of liquids; and, finally, when they were named; and that they resembled, but in a greatly exaggerated degree, the convulsive, sighing, respiration, occasioned by sudden immersion in cold water. The eyes assumed a staring expression, with the pupils greatly dilated. The expression indicated intense agitation and agony. The face became turgid and livid. These symptoms continued, more or less severe from five minutes to nearly half an hour. The intellect of the patient was clear; and the paroxysms seemed to be shortened by soothing management—encouraging him to exert himself to keep quiet, telling him he would injure himself, &c.

He succeeded in taking a little solid nutriment, but with great difficulty; tongue was white; fauces slightly reddened; sweating profuse; pain in arm, severe—complained throughout the disease of a benumbed sensation in the arm, but the senisbility and the power of motion were not impaired. The cicatrix of the wounds had a dull, leaden colour. The respiration in the intervals was suspirious. Tendons of feet rigid. He complained constantly of intense thirst, but said he could not bear to think of drink. He attempted to suck an orange, but on receiving the juice into his mouth, spasms were excited which prevented him from swallowing it. He was observed to gape frequently. His mind was very irritable ; and he was constantly in motion, changing his position, pulling the bed clothes, &c. His countenance had an indescribable wildness of expression.

The treatment directed on the evening of the 23d, was as follows:—℞. Proto-chlor. Hyd. grs. xx. ; Sulph. morph. grs. i. ; P. Dov. grs. xx. ; m. ft. Pulv. iv. ; one every four hours. ℞. Ung. Hyd. ℥i. ; Prot. iodide Hyd. ℈i. ;—Rub into axilla and arm. Emp. Cath. to cervical and upper dorsal region.

24th. Had taken three of the above powders during the night. The paroxysms had recurred as before, when he saw liquids, and finally without. Continued conscious until three or four this morning, and died tranquilly, at 6½, A. M. Made frequent efforts to vomit during the night, accompanied with severe spasms. His friend had remarked that he looked strangely on the afternoon of the day when he gave up work, and he took no drink after that time. It is to be added, that tenderness existed, at the first, over the cervical vertebræ, which led to the cupping on the 23d.

The utmost persuasion could not induce his friends to permit an autopsical examination.—*Buffalo Med. Journ.*

